# Cancer cell targeting by CAR-T cells: A matter of stemness

**DOI:** 10.3389/fmmed.2022.1055028

**Published:** 2022-12-13

**Authors:** Caterina D’Accardo, Gaetana Porcelli, Laura Rosa Mangiapane, Chiara Modica, Vincenzo Davide Pantina, Narges Roozafzay, Simone Di Franco, Miriam Gaggianesi, Veronica Veschi, Melania Lo Iacono, Matilde Todaro, Alice Turdo, Giorgio Stassi

**Affiliations:** ^1^ Department of Health Promotion, Mother and Child Care, Internal Medicine and Medical Specialties (PROMISE), University of Palermo, Palermo, Italy; ^2^ Department of Surgical, Oncological and Stomatological Sciences (DICHIRONS), University of Palermo, Palermo, Italy

**Keywords:** CAR-T cell therapy, immunotherapy, cancer stem cells, anti-cancer therapy, tumor microenvironment

## Abstract

Chimeric antigen receptor (CAR)-T cell therapy represents one of the most innovative immunotherapy approaches. The encouraging results achieved by CAR-T cell therapy in hematological disorders paved the way for the employment of CAR engineered T cells in different types of solid tumors. This adoptive cell therapy represents a selective and efficacious approach to eradicate tumors through the recognition of tumor-associated antigens (TAAs). Binding of engineered CAR-T cells to TAAs provokes the release of several cytokines, granzyme, and perforin that ultimately lead to cancer cells elimination and patient’s immune system boosting. Within the tumor mass a subpopulation of cancer cells, known as cancer stem cells (CSCs), plays a crucial role in drug resistance, tumor progression, and metastasis. CAR-T cell therapy has indeed been exploited to target CSCs specific antigens as an effective strategy for tumor heterogeneity disruption. Nevertheless, a barrier to the efficacy of CAR-T cell-based therapy is represented by the poor persistence of CAR-T cells into the hostile milieu of the CSCs niche, the development of resistance to single targeting antigen, changes in tumor and T cell metabolism, and the onset of severe adverse effects. CSCs resistance is corroborated by the presence of an immunosuppressive tumor microenvironment (TME), which includes stromal cells, cancer-associated fibroblasts (CAFs), tumor-associated macrophages (TAMs), myeloid-derived suppressor cells (MDSCs), and immune cells. The relationship between TME components and CSCs dampens the efficacy of CAR-T cell therapy. To overcome this challenge, the double strategy based on the use of CAR-T cell therapy in combination with chemotherapy could be crucial to evade immunosuppressive TME. Here, we summarize challenges and limitations of CAR-T cell therapy targeting CSCs, with particular emphasis on the role of TME and T cell metabolic demands.

## Introduction

CAR-T cell therapy represents a novel immunotherapeutic approach for cancer treatment. This strategy is based on the use of T cells engineered to selectively recognize a specific tumor-associated antigens (TAA) on cancer cells, overcoming the major antigens’ histocompatibility complex (MHC) restriction (Sterner and Sterner, 2021). CAR-T cells recognition of cognate antigens on cancer cells induces the activation of cytotoxic signaling, including the release of granzyme, perforin and cytokines, with consequent elimination of transformed cells (Zhao et al., 2018).

The use of CAR-T cell therapy in hematological malignancies has led to promising results, capable of encouraging the scientific community to use this approach also in solid tumors (Li F. et al., 2022; Qin et al., 2022; Scholler et al., 2022). Although the advantages mentioned above, CAR-T cell therapy shows some limitations primarily concerning severe adverse effects and the specificity of antigen expression on cancer cells.

As firstly described in leukemic patients treated with CAR-T cell therapy, the principal side effects concerned the onset of cytokine release syndrome (CRS), immune cell-associated neurologic syndrome (ICANS) and cytopenia in a considerable number of patients (Funk et al., 2022; Morris et al., 2022).

Similarly to hematologic malignancies, considerable limitations have been observed in solid tumors, such as toxicity, an heterogeneous expression of the antigen, an impaired CAR-T cell trafficking to the tumor site, antigen escape phenomena, an immunosuppressive tumor microenvironment (TME) and cancer metabolism (Di Stasi et al., 2009; Peng et al., 2010; Moon et al., 2014; Sun et al., 2018).

TME is composed by the extracellular matrix, soluble molecules, and different types of cells that surround the tumor, influencing cancer growth, dissemination, and response to immunotherapy. Moreover, the tumor metabolic rewiring induced by TME components unavoidably alters the antitumor response.

Cancer stem cells (CSCs) constitute a cell subpopulation within the tumor mass implicated in cancer progression and escape from therapy. This phenomenon is explainable by the capability of CSCs to express high levels of drug efflux pumps and anti-apoptotic proteins, to proficiently repair DNA damage, to enter in a quiescent state and most importantly to evade the immune system surveillance. Moreover, failure of anti-cancer treatments is indeed ascribable to the difficulties of standard therapies to distinguish normal stem cells (NSCs) from CSCs. Thus, an emerging priority is to develop new accurate strategies to selectively eliminate CSCs (Turdo et al., 2020; Veschi et al., 2020; Gaggianesi et al., 2021; Turdo et al., 2021).

CAR-T cells have shown the ability to recognize specific cancer stemness markers and efficiently eradicate CSCs while sparing NSCs (Masoumi et al., 2021). This evidence opens new venues in the field of immunotherapy due to the potential of CAR-T cell-based therapy to target tumor heterogeneity for an effective cancer treatment.

However, a limitation in the use of CAR-T cells is linked to the presence of shared antigens between CSCs and normal cells, determining a phenomenon known as “off-target” with the unspecific killing of non-cancerous cells. Thus, it is necessary to identify new CSC-specific antigens that can be recognized by CAR-T and potentiate their activity by combining multiple treatments including chemotherapy and targeted therapies (Wang H. et al., 2020).

In this review, we discuss the latest implementations in CAR-T cell engineering with the scope of overcoming the influence of TME components and ameliorate a specific cancer cell recognition. In addition, we dissect the most powerful therapeutic approach that target CSCs peculiarities for an effective cancer treatment.

## The structure of CAR-T cells

CAR basic design is composed of i) an extracellular domain that consists of a single-chain variable fragment (scFv) region with a light (VL) and heavy chain (VH) that recognizes tumor-associated antigens (TAAs), ii) A spacer region or hinge, iii) A transmembrane domain, which anchors CAR to the membrane of T cells and iv) An intracellular domain composed by the T cell activation and co-stimulatory domains.

It has been reported that the VH and VL domains can induce an immune reaction in patients after CAR-T infusion, by producing antibodies against scFv of CAR-T, impairing its anti-tumor effect. To overcome this obstacle the CAR extracellular domain was manipulated in order to insert a single variable domain on a heavy chain (VHH), also called nanobody. As scFv, the nanobodies owe the same properties regarding binding affinity, specificity, stability, and solubility after CAR-T infusion in patients (Safarzadeh Kozani et al., 2022a).

An important advantage of CARs compared to the conventional TCRs is the possibility to act in an human leucocytes antigen (HLA) independent way (Brownlie and Zamoyska, 2013). The interaction between CAR binding domain and the specific antigen, expressed on tumor cells, induces the activation of ζ or γ chains cross-linking that form the chimeric receptor intracellular domain. Then, T cells lytic pathway is activated *via* the releasing of cytosolic granules containing granzymes and perforin. Perforins multimerize on target cell surface and form pores that facilitate the movement of granzymes into the host cells. The final result of this immune response is the activation of the apoptotic cell death program in the targeted cells (Boivin et al., 2009).

In order to improve the T cell signaling, different generations of CAR have been developed over the years by modulating the intracellular domains. The oldest version of CARs presented one cytoplasmic CD3 signaling domain, which allowed a mild activation of T cells due to exhaustion and anergy of CAR-T cells.

For these reasons, the 2nd generation of CAR was engineered by adding one co-stimulatory domain among CD28, 4-1BB (CD137), OX40 or ICOS. This intracellular complex was more efficient in inducing a greater T cell response in terms of cytokines production and expansion rate *in vivo* (Liu et al., 2016; Zhao et al., 2018; Rafiq et al., 2020; Mehrabadi et al., 2022). The combination of multiple costimulatory domains, such as CD3-C28-4-1BB or CD3^−^CD28-OX40, allowed the development of the 3rd generation of CAR characterized by increased anti-tumor activity and persistence of T cells. Based on the 3rd generation of CAR, the 4th generation also called TRUCKs (T cells redirected for antigen-unrestricted cytokine-initiated killing) has been developed to express different proinflammatory cytokines [such as interleukin (IL)-2, IL-5, IL-12, IL-15 and IL-17] in addition to CD3 domain. Following the binding between the CAR and the tumor antigen, at the CAR-T cell intracellular domain, nuclear factor of activated T cell (NFAT) is phosphorylated and, *via* translocation to the nucleus, induces the expression of the transgenic cytokines. The released cytokines have a dual effect: autocrine because they support T cells in terms of survival and proliferation, and paracrine by modulating the immune microenvironment present at the tumor site. The TRUCK strategy boosts CAR-T cell resistance to the immunosuppressive TME and leads to the recruitment of immune system cells to the tumor site (Dragon et al., 2020). The last generation, the fifth, includes in addition to CD3 and a costimulatory molecule (CD28 or 4-1BB), a truncated intracellular IL-2Rβ domain with a STAT3 binding motif. The link between the CAR and the cognate tumor antigen, causes the IL-2Rβ-mediated activation of the JAK/STAT pathway with consequent proliferation and persistence of CAR-T cells (Tokarew et al., 2019; Xin et al., 2022).

A feature shared by the last two generations of CAR-T is the ability to mitigate the systemic release of cytokines and to induce pro-inflammatory cytokines release only after contact between the T cell and the tumor cell, thus reducing systemic toxicity and the CRS (Rafiq et al., 2020; Tian et al., 2020; Mehrabadi et al., 2022).

In the last years, numerous efforts have been made by the scientific community to better engineer CAR-T cells. In particular, one of the major hurdle to be faced is the loss or downregulation of the TAAs in cancer cells, leading to the failure of CAR-T cell therapy (Sterner and Sterner, 2021; Mehrabadi et al., 2022). Thus, in order to enhance the CSCs killing and avoid the antigen escape phenomenon, it would be necessary to combine different therapeutic approaches aimed at targeting multiple tumor features, such as CAR-T cell-based therapy in combination with monoclonal antibodies or with chemotherapy.

## Properties of cancer stem cells (CSCs)

Failure of standard therapies against tumors probably depends on the presence of many clonal cell populations, differing at genetic and phenotype level, that compose tumor mass. In particular, the presence of tumor cell subpopulations with stemness features, appears to be fundamental to confer refractoriness to therapies (Bonnet and Dick, 1997; Reya et al., 2001).

CSCs have been identified for the first time by Dick et al., in a liquid tumor, the acute myeloid leukemia (AML), subsequently, CSCs have been found in many solid tumors, such as colon, breast, lung, melanoma, and pancreatic cancer (Bonnet and Dick, 1997; Batlle and Clevers, 2017). The CSCs subpopulation is considered the seed responsible for tumor initiation and progression. One of the most accredited hypotheses by the scientific community is that CSCs derive from NSCs that populate adult tissues (Shackleton, 2010; Rossi et al., 2020). When NSCs acquire genetic and epigenetic alterations, they lose their genome integrity, undergo deregulation of signaling pathways, and achieve a malignant phenotype (Verona et al., 2022). CSCs are characterized by self-renewal, asymmetrical division capability, active telomerase expression and anti-apoptotic pathway, activated DNA repair machinery, an unlimited proliferative capacity, and a high number of ABC transporters for drug efflux (Rossi et al., 2020). In fact, it has been demonstrated that CSCs are able to generate a solid tumor within heterogeneous cancer cells in immunodeficient mouse models (Al-Hajj et al., 2003; Todaro et al., 2014). Moreover, CSCs are responsible for tumor recurrence and standard therapies resistance also due to their dormant status (Li et al., 2008; Talukdar et al., 2019). Therefore, targeting CSCs represents a challenge to fight cancer.

**FIGURE 1:**
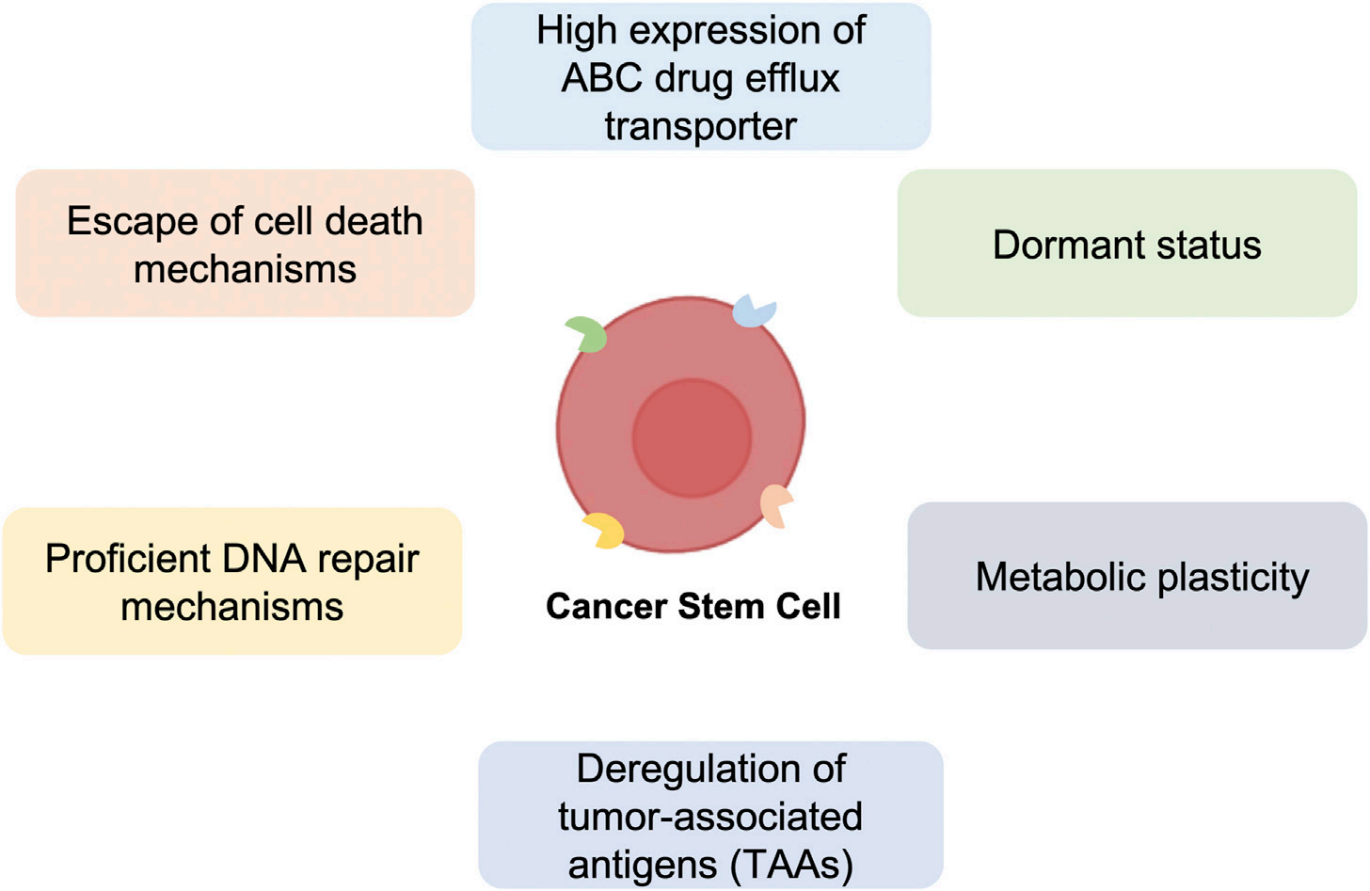
Major mechanisms of anti-cancer therapy resistance of Cancer Stem Cells (CSCs). Schematic representation of the principal Cancer Stem Cells (CSCs) characteristics, including high expression of ABC drug efflux transporter, dormant status, metabolic plasticity, deregulation of tumor-associated antigens (TAAs), proficient DNA repair and escape of cell death mechanisms, which confer resistance to standard anti-cancer therapies.

Since CSCs were discovered, it has been necessary to isolate this population to investigate their features and role. This process is carried out through the characterization of the CSCs surface molecules (Huang et al., 2022). Examples of CSCs markers are CD19 (Hosen, 2013; Zhao et al., 2017), CD34 (Aravindan et al., 2021), CD44 (Al-Hajj et al., 2003; Zhang et al., 2008), CD44v6 (Todaro et al., 2014), CD54 (Chen et al., 2012), EpCAM (Osta et al., 2004), CD114 (Hsu et al., 2013), CD117 (Sundberg et al., 2009), CD133 (Bach et al., 2013), CD271 (Boiko et al., 2010), and ALDH (Ginestier et al., 2007) (Figure 1).

However, the identification of CSCs markers is not easily feasible, because these are often expressed also on NSCs and normal tissue. Therefore, variable panels of markers are used to identify the tumor stem cell populations for each type of cancer. Markers used for the CSCs isolation are also used for the formulation of new therapeutic approaches, such as targeted- and CAR-T cell therapy. Therefore, continuing to deepen the knowledge of the CSCs’ role in cancer development and the identification of specific CSCs surface markers, easily recognized and detected by CAR-T cells, will allow the implementation of more effective CAR-T cell-based therapies.

## Tumor microenvironment: Limitations and opportunities

A favorable microenvironment is required for a stronger anti-tumor immune response. Tumors are an elaborate ecosystem in which cancer cells and normal cells coexist in an enriched and unique extracellular space. The contribution of the tumor landscape is becoming increasingly relevant to define a successful anti-tumor therapy (Gkretsi et al., 2015; Jin and Jin, 2020; Wei et al., 2020; Di Franco et al., 2021).

It is known that CSCs have a continuous interaction with the components of solid TME, such as macrophages, cancer associated fibroblasts (CAFs), immune, endothelial and adipose cells, and extracellular components (Gaggianesi et al., 2021; Belli et al., 2022). CSCs and TME components regulate each other in a feedback loop during all the phases of tumorigenesis, cancer promotion and progression. Stromal cells are recruited and re-educated by CSCs to produce pro-tumoral cytokines, growth factors and peptides that create a pro-inflammatory and immune suppressive environment favorable for tumor growth (Barcellos-Hoff et al., 2013).

CSCs orchestrate the formation of a protective shield against the external environment to evade the anti-tumor immune system response and in addition, to escape anti-cancer therapies, such as CAR-T cell therapy (Gaggianesi et al., 2021; Yang et al., 2021).

Adoptive immunotherapy, by engineering T cells with a specific CAR, is profoundly affected by the presence of an adverse microenvironment. Indeed, CAR-T cells do not act in a strictly cell-autonomous way, but through a complex crosstalk with the TME machinery (Marofi et al., 2021; Sterner and Sterner, 2021).

The presence of a dense fibrotic mass in the solid tumor, a low quantity of chemokines involved in the recruitment of lymphocytes, reduces the migratory and penetrative capacity of CAR-T cells (Li et al., 2018). Moreover, T cell infiltrating activity is negatively modulated by extracellular purine nucleosides, such as adenosine (ADN) (Ohta, 2016; Boison and Yegutkin, 2019). There are four known ADN receptors, A1, A2a, A2b, and A3 receptors. The A2a receptor (A2aR) is present on the surface of T lymphocytes. Binding of ADN to its own receptor on the CD4^+^/CD8^+^ cell surface reduces interferon- γ (IFN-γ) and granzyme B production (Sorrentino et al., 2019). For this reason, different methods have been developed to inhibit A2aR and to increase the activity of T cells by overcoming the inhibition caused by ADN in the hostile microenvironment (Fallah-Mehrjardi et al., 2020). SCH-58261 is an A2aR antagonist, but its inhibitory activity is hampered by poor drug solubility and pharmacokinetic properties *in vivo*. To overcome this limitation, Siriwon et al., generated engineered CAR-T cells expressing an encapsulated vesicle containing SCH-58261, allowing drug delivery at the tumor site. These results demonstrated that conjugating, *ex vivo* before the administration, liposome multilamellar drug-loaded nanoparticles on the CAR-T cell’s surface, augments significantly the efficacy of CAR-T therapy reducing the delivery to other tissue sites thus minimizing side effect phenomena (Siriwon et al., 2018). Moreover, Masoumi et colleagues, demonstrated that associating a gene silencing system (shRNA) against the A2aR receptor, in the human anti-mesothelin CAR construct, could increase CAR-T cell therapy efficacy in solid tumors (Masoumi et al., 2020).

Tumor-associated macrophages (TAMs) are the predominant cells of TME that contribute to tumor progression and hamper immune response by a direct effect on cancer cells (Yu et al., 2021). M2-like TAM exploit their immune suppressive function by anti-inflammatory cytokines production, the expression of immune checkpoint ligands, and the release of immunomodulatory enzymes such as arginase I, which is involved in the arginine degradation essential for T cell functions (Sharda et al., 2011; Sosnowska et al., 2021). CSCs are responsible for TAMs re-education to support tumor growth. To redirect macrophage phenotype from M2 (pro-tumorigenic phenotype) toward M1 (anti-tumorigenic phenotype), several strategies have been described consisting on the use of CD40 antagonists, PI3K inhibitors, or antibodies against the CCR2 ligand (Pathria et al., 2019). M2-like TAMs secrete IL-6 that induces STAT3 signaling activation in CSCs promote their survival and enhance their proliferation (Radharani et al., 2022). In pancreatic cancer, the depletion of M2 macrophages reduced stem cell tumor compartment improving response to chemotherapy. Rodriguez-Garcia et al., following the identification of folate receptor β (FRβ) as a specific marker for the M2 TAM subtype, generated CAR-T cells directed against this surface receptor. Targeting FRβ positive TAMs in melanoma syngeneic mouse models resulted in a switch of TME increasing the homing and activity of T cells and the recruitment of monocyte with anti-tumor activity. Tumor pre-treatment with FRβ CAR T cells may, thus, increase the anti-tumor activity of adoptive T cells therapy (Rodriguez-Garcia et al., 2021).

CSCs and stromal cells recruited at the tumor site promotes an inflammation state that exacerbates tumor progression by an overexpression of cytokines and their receptors (Vlasova-St Louis and Bohjanen, 2017; Lopez de Andres et al., 2020; Quinn et al., 2020). In many solid tumors, the cytokine-chemokine network is profoundly altered by the cross-talk between CSCs and other stromal cells, with a consequent release of soluble factors that favor an immune suppressive condition (Chen W. et al., 2018; Reshkin and Cardone, 2020).

Cytokines and chemokines are small secreted molecules that interfere with the survival, expansion, homing, differentiation, and activity of T cells (Borish and Steinke, 2003). Among the immunosuppressive cytokines, IL-4, TGFβ and IL-10 are the most representative at the tumor site, contributing to CAR-T cell dysfunction (Ghahri-Saremi et al., 2021). To circumvent this limitation Mohammed et al., generated a unique protein in which the cytokine-binding domain of the IL-4 receptor was fused with the endodomain of the IL-7 receptor involved in the immunostimulatory signaling (Mohammed et al., 2017). The infusion of CAR-T cells against a prostate-specific antigen engineered to express the inverted cytokine receptor resulted in potent and sustained anti-tumor effects (Mohammed et al., 2017). On the contrary same efforts have been made to force the expression of pro-immune cytokines generating “Armored” CAR-T cells that are engineered to secrete pro-inflammatory cytokines such as IL-12, IL-15, and IL-18 (Jorgovanovic et al., 2020).

The non-cellular components of TME such as tenascin, periostin, SPARC, and collagen which overall contribute to the fitness of the tumor tissue, are mainly released by CAFs (Chiquet-Ehrismann et al., 1986). A plethora of neoplastic tissues, including pancreatic cancer, are characterized by a dense desmoplastic reaction in which a large amount of extracellular components strongly supports tumor growth and limits drug penetration and uptake (Modica et al., 2021). The presence of a dense stroma compartment negatively influences CAR-T cells therapy limiting homing at the tumor site, downregulating the expression of adhesion molecules, and acting as a reservoir for immunosuppressive cytokines (Gorchs and Kaipe, 2021). In the CSCs compartment, CAFs play an important role through IGF-II/IGF1R pathway and Wnt signaling cascade sustaining CSCs survival (Valenti et al., 2017). Moreover, prostaglandin E2 (PGE2), produced by CAFs, is directed against CSCs compartment in which activates NF-κB, *via* EP4-PI3K and EP4-mitogen-activated protein kinase signaling, promoting CSCs expansion (Wang et al., 2015).

For instance, targeting CAF-derived factors such as PGE2, may represent a strategy to weaken the CSCs compartment and enhance immunotherapy efficacy in solid tumors (Wang et al., 2015; Freeman and Mielgo, 2020; Safarzadeh Kozani et al., 2022b). Conversely, Sakemura and others have developed a strategy that directly acts on activated fibroblast by engineering CAR-T cells to target both multiple myeloma cells and signaling lymphocyte activation molecule family-7 (SLAM-7) expressed in CAFs. The dual targeting of stroma components and cancer cells, enhancing CAR-T cell cytotoxicity activity and overcoming resistance to the anti-tumor adoptive cell therapy, represents an encouraging therapeutic approach (Sakemura et al., 2022).

TME is also characterized by the presence of a degenerate vasculature consisting of vessels of abnormal size, tortuous, with oversized pores, and without pericytes to cover the structure. The corrupted vasculature results in a dysfunctional blood flux and low oxygen level responsible for the hypoxic condition observed in solid tumors. In addition, the aberrant blood flux is also responsible for the accumulation at the tumor site of metabolites and toxins (Jain, 2005).

T cells activity is hampered not only by a physical barrier, due to abnormalities of the vessels, but also by tumor-associated endothelial cells that promote an immunosuppressive environment by downregulation of adhesion molecules. Intracellular adhesion molecule 1 (ICAM1) and vascular cell adhesion molecule 1 (VCAM1) are dramatically reduced in tumor vessels determining a critical obstacle for T cells extravasation. On the contrary, the low expression level of adhesion molecules promotes FoxP3^+^Treg accumulation exacerbating the immunosuppressive environment (Terme et al., 2013). Moreover, their suppression activity is boosted by CD39 which catalyzes the hydrolysis of ATP into ADN and contributes to FoxP3^+^Treg stability (Takenaka et al., 2016).

Bevacizumab, a monoclonal antibody directed against VEGF, has been approved for the treatment of several solid tumors inducing a re-modulation of tumor vessels, increasing B and T cell recruitment, and improving immune response (Roviello et al., 2017). The combination treatment of CAR-T cells with anti-VEGF molecule resulted a significant increment in immunotherapy efficacy (Bocca et al., 2017). T cell treatment of solid tumors has proven to be a challenge, largely due to the hostile solid TME. One of the active and cellular TME components is represented by myeloid derived suppressor cells (MDSCs). They mainly act in the peripheral blood and at the TME site where they foster an innate and adaptive anti-immune system response. The interplay between MDSCs and tumor stroma is in charge of structural and functional modification of the TME. MDSCs are in close contact to cellular and non-cellular microenvironmental components, they influence the blood vessel’s morphology and functionality and interact with CSCs enhancing tumor invasion and metastasis formation. More importantly, MDSCs settle positive feedback loop with CSCs. MDSCs are recruited by CSCs through G-CSF at the tumor site where in turn they prompt CSCs stemness properties *via* NOTCH/STAT3 signaling cascade (Welte et al., 2016; Ouzounova et al., 2017). In TME, MDSCs rewire their metabolism significantly increasing metabolites uptake (such as glucose, fatty acid, lactate and aminoacid) defining an immunosuppressive microenvironment (Wang Y. et al., 2020). Blocking the immunosuppressive role of MDSCs, may boost the efficacy of CAR-T cell treatment (Holthof et al., 2021).

An important strategy based on the use of conjugated CAR-T cells is represented by engineering the R2.4-1BB of the TR2 receptor. This receptor class is physiologically expressed on the TME-resident MDSCs, leading to their suppression and lack of function. CAR-R2.4-1BB engineered structure, lead to anti-immunity inhibition and augments intratumoral CAR-T proliferation and clinical potential of CAR-T cell therapy in solid tumors (Nalawade et al., 2021).

**FIGURE 2:**
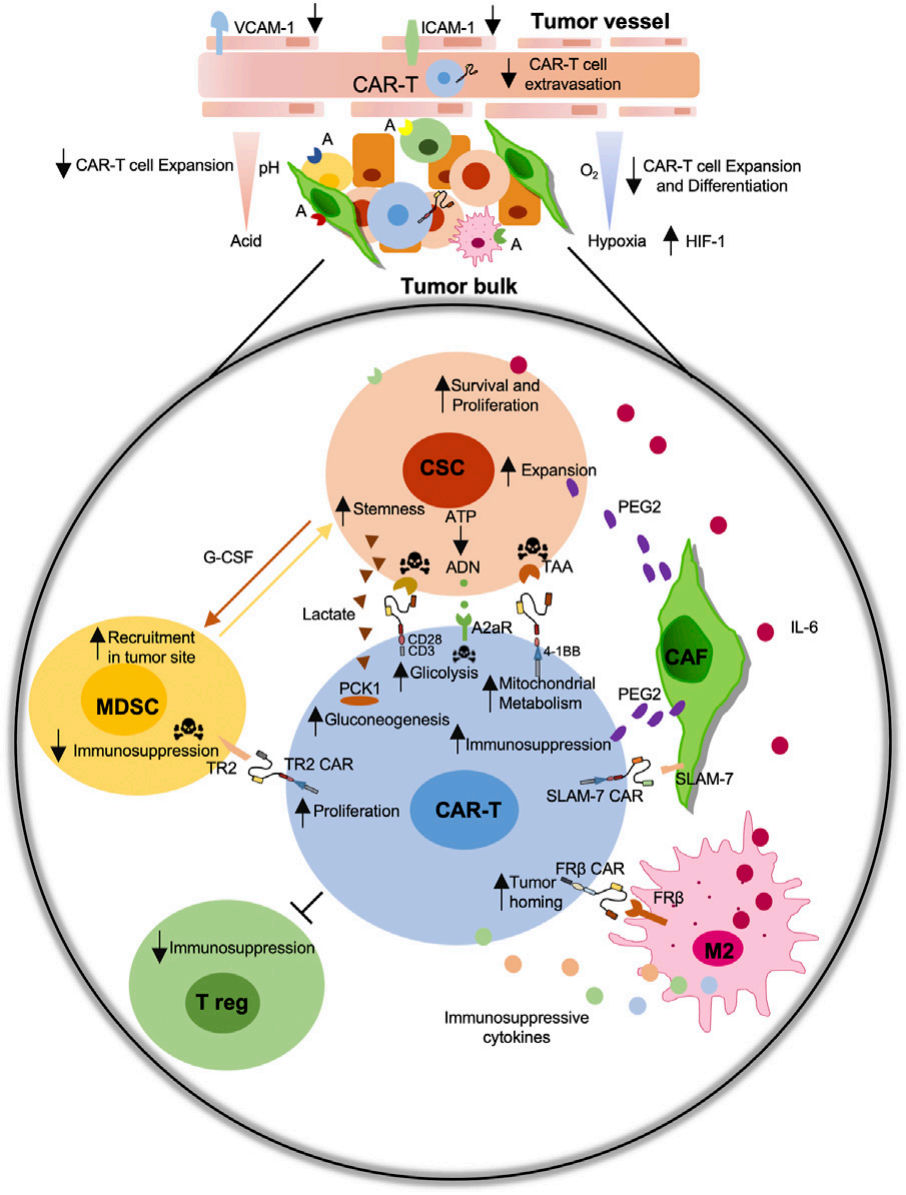
Schematic representation of the immunosuppressive role of the tumor microenvironment (TME) on the CAR-T cell efficiency. Immunosuppressive tumor microenvironment (TME) including cancer-associated fibroblast (CAFs), M2-macrophages, myeloid-derived suppressor cells (MDSCs), regulatory T cells (Treg) negatively affect CAR-T cell activity against Cancer Stem Cells (CSCs). The development of CAR-T cell-based strategies against CSC-specific tumor associated antigens (TAAs), including EpCAM, CD44v6, CD166, c-Met, CD133, or against specific TME components antigens (A) including TR2, FRβ, SLAM-7 improve their functions.

In conclusion, the active role played by TME and the possibility to modify its structure and cell components make it one of the most important challenges to increase the efficacy of CAR-T-based treatments (Figure 2). Thus, the possibility to create therapies directing CAR-T cells against TME modulator is a promising prospect in tumor immunotherapy treatment.

## Cancer stem cell metabolic rewiring: A pivotal barrier for CAR-T

The metabolic profiles of CSCs, including all factors released in the TME, are responsible for immunotherapy efficiency and resistance (Chew et al., 2012). CSCs must fulfill the energy demands for a strong synthesis of metabolites, which boosts uncontrolled proliferation. For these reasons, cancer cells, including CSCs, have a deregulated metabolism, a common feature of all cancer types. According to the theory formulated by Otto Warburg, in 1927, it is believed that the tumor mass has a purely fermentative glycolytic metabolism, which allows the conversion of glucose into pyruvate and also, in aerobic conditions, into lactate. In addition, glycolysis is essential for the production of metabolic intermediates, and glucose-6-phosphate is used for the synthesis of nucleotides (Warburg et al., 1927; Kroemer and Pouyssegur, 2008; Turdo et al., 2020).

Many studies have subsequently shown that the tumor mass is heterogeneous, also on the metabolic aspect. CSCs have an intrinsic plastic metabolism to suit unfavorable conditions, converting a hostile environment into a favorable milieu for their survival and progression (Janiszewska et al., 2012; Sancho et al., 2016; Chae and Kim, 2018; Mori et al., 2019; Emami Nejad et al., 2021).

Cancer cell demand of intermediate metabolites as well as the adverse microenvironmental conditions dictate changes in metabolism also in CSCs subpopulation. For instance, a mitochondrial metabolism allows CSCs to develop numerous intermediate metabolites produced by the Krebs cycle and by pathways connected to it, such as fatty acid metabolism. Furthermore, the rapid metabolic change represents an escape mechanism implemented by CSCs to standard therapies (Turdo et al., 2020). This exaggerated demand leads to nutrient competition of all TME components, including lymphocytes (Chang et al., 2015). PD-L1 *via* PI3k/mTOR pathway plays a critical role in regulating CSCs glucose uptake (Hudson et al., 2020). Pembrolizumab, nivolumab, and cemiplimab, by blocking the PD-1/PD-L1 axis, reduce glucose uptake by cancer cells, leading to an increment of TME glucose concentration that is necessary for CAR-T cell activation (Xu et al., 2019).

Cancer cells adapt their oxygen supply requirement to survive and grow, as well as stemness compartment. Hypoxia represents one of the most frequent hostile conditions to which CSCs are subjected during tumor promotion and growth. In the hypoxic conditions, cells undergo a cell cycle block, entering in a quiescence state that reduces their susceptibility to conventional therapy (Das et al., 2008; Ju et al., 2022). Therefore, the identification of the escape mechanisms of CSCs from hostile conditions can allow the development of artificial systems that help in the fight against these important subpopulations.

## Optimization of CAR-T cell metabolism

T lymphocytes have a baseline metabolism of oxidative phosphorylation and the oxidation of fatty acids (FAO). During the activation process, based on the surrounding environment and the type of antigen that has been encountered, the lymphocytes proceed to differentiate with regulatory or effector functions. Several authors show that metabolism is a key element in regulating the proliferation and differentiation of T cells (Kaech et al., 2002; Chang et al., 2013; Chang et al., 2014; Kouidhi et al., 2018; Gutierrez-Arcelus et al., 2019).

T lymphocytes reprogram their metabolism in line with their needs (Donnelly and Finlay, 2015). One of the most activated pathways in effector cells is the PI3K-AKT-mTOR, which converges in the activation of c-Myc, the overexpression of type 1 glucose transporters (GLUT1), greater glucose uptake, and the promotion of aerobic glycolysis. Subsequently, it was also observed that memory cells exhibit increased oxidative phosphorylation (Frauwirth et al., 2002; Wang et al., 2011; Zhang et al., 2021).

The regulatory T cells instead show a metabolism based more on the FAO (Ma et al., 2019). When T cells are taken from the patient, to be engineered with a CAR construct, they must undergo *in vitro* purification and expansion processes. During and after the *in vitro* expansion process, the choice of medium is also crucial. The use of nutrient-rich media (carbohydrates and amino acids) is required to generate sufficient CAR-T for infusion. However, *ex vivo* culture conditions characterized by excessive amounts of nutrients can compromise the survival of CAR-T when infused in patients. Indeed, the presence of a hostile environment and low glucose provokes an early exhausted T cell phenotype, responsible for the failure of immunotherapy. In addition, glucose is required for T cell activity to produce proinflammatory cytokines, such as IFN-γ. Therefore, accustoming CAR-T cells to a low glucose environment, resulting in a gradual reduction in glycolysis, will allow a higher success rate (Turdo et al., 2020; Kankeu Fonkoua et al., 2022).

Another important precaution that must be taken during the expansion phase of engineered T lymphocytes concerns the pH of the medium in which they grow. According to Rodrigo Lamas et colleagues, even small changes in pH can alter the growth and functioning of CAR-T cells. An acid pH between 6.8 and 7.0 counteract the expansion process, while a slightly basic pH of 7.5 supports robust expansion (Lamas et al., 2022).

Cytokines added in culture media during the expansion phase are also fundamental element that may enhance cell proliferation and differentiation. For example, IL-2 promotes glycolysis, IL-7 actives the STAT5 pathway and glucose uptake, IL-15 increases stability of mitochondrial health and down regulates the glycolysis pathway (Wofford et al., 2008; Secinaro et al., 2018).

In order to successfully reach tumor bulk, lymphocytes must be able to survive in the TME and preserve their faculties by adapting their metabolism (Beckermann et al., 2017; Ghassemi et al., 2020). In the microenvironment, there are a plethora of metabolic factors which act as immunosuppressive elements and inhibit effector T lymphocyte activity.

Lactate is generated by hyperproliferating tumor cells in presence of an inadequate tumor vascularization. The presence of lactate in TME influence negatively T cell functions leading to anti-tumor response inefficacy by preventing the activation of cytotoxic T lymphocytes and dendritic cells (DeBerardinis and Chandel, 2016). The metabolism of CAR-T *in vitro* also changes according to the different co-stimulating domains used in the construct (CD28, ICOS, 4-1BB, OX40 or CD27) (Maus et al., 2013; Liu et al., 2016). For instance, CD28 enhances the glycolytic pathway, reduces cell proliferation and secretome, and leads T cell effectors to a rapid exhaustion. Instead, 4-1BB sustains mitochondrial oxidative metabolism that boosts cell persistence *in vivo* (Pellegrino et al., 2020). In accordance to the above mentioned role of mitochondrial metabolism, Chowdhury PS et colleagues observed a better and prolonged anti-tumor performance of T cells in MC38-bearing mouse treated with PD1 blockade therapy by improving the mitochondrial activity of T cell (Chowdhury et al., 2018; Li and Zhang, 2020).

Interestingly, Zhao et al. compared 4-1BB-based CAR-T to CD28-expressing CAR-T cells. In particular, 4-1BB CAR-T cells owned a higher anti-tumor activity and longer persistence in NCG mice engrafted with the Daudi, NALM6, Raji, and K562 leukemia cell lines. Furthermore, through a retrospective analysis, the performance of the two different CAR-T cells has been examined on thirty-six patients. The retrospective study showed that patients infused with the 4-1BB CAR-T cells showed a higher overall survival rate and less severe adverse events as compared to CD28 CAR-T infused in patients (Zhao et al., 2020). In addition, CAR constructs formed by the costimulatory proteins OX40, CD27, and ICOS have been subject of extensive studies (Peperzak et al., 2010; Zeng et al., 2016; Pacella et al., 2018; Weinkove et al., 2019). Non-etheless, it has been demonstrated that OX40 determines the regulation of glucose and lipid metabolism while CD27 appears to be involved in the regulation of oxidative stress and glycolysis. Conversely, ICOS allows a greater activity of GLUT-1 and lipid synthesis (Peperzak et al., 2010; Zeng et al., 2016; Pacella et al., 2018; Weinkove et al., 2019). As suggested by Kawalekar et al., a promising therapeutic approach that recapitulates the natural immune response could be to combine CAR-T cells created with the CD28 domain, composed mainly of effector T lymphocytes with glycolytic metabolism, with the CAR-T created with the 4-1BB domain composed mainly of memory T cells bearing a mitochondrial metabolism (Kawalekar et al., 2016).

Careful and in-depth studies are needed to choose the best CAR construct in accordance to the tumor type and the availability of oxygen in the TME. Indeed, different metabolic pathways have different oxygen requirements (Zhang et al., 2007; Teijeira et al., 2018). In solid tumors, low levels of oxygen induce TME cells to activate the hypoxia pathway. In a mouse model recapitulating a solid tumor, T cells have shown a stabilization of the hypoxia-inducible factor (HIF-1) with consequent rewiring of their metabolism, promoting anti-cancer activities (Rodriguez-Garcia et al., 2020). Recent studies showed the possibility to exploit the hypoxic microenvironment using CAR-T engineered for the oxygen sensible domain of HIF-1A, which improve the CAR-T metabolism and function in low oxygen concentration (Xu et al., 2019).

Considering the central role of metabolism in the activity, survival, and success of CAR-T in anti-cancer treatments, full knowledge about cell metabolism, could be a key point for improving the immune response of CAR-T cell therapy (Figure 2).

## Targeting cancer stem cells by CAR-T cells in pre-clinical and clinical studies

In the last few years, the application of CAR-T cell therapy targeting CSCs has obtained remarkable success in the treatment of several hematologic tumors, but poor results have been achieved in targeting malignant solid tumors. It has been demonstrated that CAR-T cell monotherapy is not sufficient for the complete elimination of CSCs in solid tumors, indicating the necessity to combine it with other therapeutic approaches (Maiuthed et al., 2018; Han D. et al., 2021). CAR-T cell therapy efficacy is hindered by several factors such as the immunosuppressive microenvironment, tumor heterogeneity and CSCs plasticity and immune escape capacity (Gilham et al., 2012; Marofi et al., 2021).

One additional adoptive T cell therapy limitation is the “on-target off-tumor toxicity” resulting in the killing of NSCs that share the same targeted antigens expression to CSCs. Another adverse event is associated with the release of excessive cytokines which cause the so-called CRS. This toxicity could be minimized by introducing suicidal genes, like the inducible caspase 9, that can induce apoptosis of T cells, preventing their over-activation (Gargett and Brown, 2014).

### EpCAM

EpCAM is a type I transmembrane glycoprotein, mainly involved in cell proliferation, migration, differentiation, and cell adhesion. Deng et al. generated EpCAM CAR-T cells to specifically target the metastatic prostate cancer cells (PC3M) that express high levels of the CSC antigen EpCAM. By performing both *in vitro* and *in vivo* experiments, using NOD/SCID mice, the authors demonstrated that EpCAM CAR-T cells killed EpCAM-overexpressing PC3M cells. Moreover, EpCAM CAR-T cells suppressed growth and the metastatic capacity of PC3 parent cells expressing low EpCAM levels (Deng et al., 2015). Recently, Zhang et al. used third-generation CAR-T cells specific to EpCAM. This study revealed that EpCAM CAR-T cells secreted cytotoxic cytokines, like tumor necrosis factor-alpha (TNF-α) and IFN-γ and delayed the cancer growth in xenograft models, showing no toxicity in mice (Zhang et al., 2019). Several clinical trials are still ongoing in which EpCAM CAR-T cell’s inhibitory activity is under evaluation alone or in combination with chemotherapy for the treatment of many solid tumors (NCT02915445; NCT03563326; NCT03013712; NCT02729493; NCT02725125).

### CD44

CD44 is a transmembrane receptor expressed on the surfaces of CSCs of different tumor types that binds hyaluronic acid, regulating cell-cell and cell-matrix adhesion. It is also involved in the epithelial-mesenchymal transition and cell proliferation (Dalerba et al., 2007; Prince et al., 2007; Lee et al., 2008). Our research group has shown that the v6 variant of CD44 is a key factor in the migration, metastasis, and resistance to target therapy of colorectal cancers (Todaro et al., 2014). Engineered T cells targeting CD44v6 (CD44v6 CAR-T) showed an anti-tumor effect *in vitro* and *in vivo* in various cancers such as acute myeloid leukemia, multiple myeloma, and pulmonary and ovarian adenocarcinoma (Casucci et al., 2013; Porcellini et al., 2020). Currently, the use of CD44v6 CAR-T is ongoing in phase 2 clinical trials in different types of cancer (NCT04427449).

### CD166 or ALCAM

Another CSCs surface marker is CD166 or ALCAM (activated leukocyte cell adhesion molecule), a transmembrane glycoprotein that belongs to the immunoglobulin superfamily (Dalerba et al., 2007; Jiao et al., 2012; Yan et al., 2013). Physiologically, it regulates hematopoiesis, neurogenesis and inflammatory responses but it is also highly expressed and associated with tumorigenesis in many different tumors, such as breast, colorectal, prostate, melanoma, and pancreatic cancers (King et al., 2004; Federman et al., 2012). In a recent study, CD166-specific CAR-T cells were tested *in vitro*, in MNNG/HOS, U2OS, MG-63 and Saos-2 osteosarcoma cell lines, and *in vivo*, in NOD/SCID mice, in order to evaluate the capability of CAR-T to selectively target CD166^+^ cells. Of note, CD166 CAR-T cells hampered tumor growth without injury against healthy tissues. These data support the use of CAR-T also in other CD166 expressing tumors with immunotherapy and/or chemotherapy (Wang et al., 2019).

### c-Met

c-Met is a tyrosine kinase receptor, a proto-oncogene expressed in both cancer and normal cells, activated by its ligand, the hepatocyte growth factor (HGF). It promotes a wide range of activities in cancer, such as angiogenesis, tumor growth, cell motility, and metastasis. c-Met activation in cancer occurs by overexpression, mutations, and amplification of the gene (Boccaccio and Comoglio, 2006). Recent studies have shown that c-Met is a putative stem/progenitor cell marker in colorectal cancer, glioblastoma, and breast cancer (Di Renzo et al., 1991; Li et al., 2011; Baccelli et al., 2013; Lin et al., 2019). Recently, Kang et al. demonstrated that the c-Met CAR-T cells co-cultured with c-Met-positive gastric cancer cells secreted IL-2 and IFN-γ, showing specific anti-cancer cytotoxicity. Moreover, the c-Met CAR-T cells suppressed tumor growth *in vivo* xenograft models, without any significant side effects in mice (Kang et al., 2021). Of note, a phase 1 of clinical trials were terminated using autologous T cells engineered with c-Met in breast cancer (NCT01837602). The pharmacological treatment with autologous T cells engineered with c-Met in metastatic breast cancer patients, showed no side effects after intratumoral injections. Interestingly, after resection of tumors, at the injection site, it has been observed a considerable necrotic area with macrophages infiltration (Tchou et al., 2017).

### CD133

CD133, or prominin-1, is a pentaspan transmembrane glycoprotein, encoded in humans by the *PROM1* gene. It is a CSCs marker in glioblastoma, colorectal, liver, and pancreatic cancer (Liu et al., 2006; Hermann et al., 2007; Ma et al., 2008; Ren et al., 2013). Wang et al. in a phase 1 trial (NCT02541370), have demonstrated that using CD133CAR-T cells in refractory and metastatic tumor patients with hepatocellular carcinoma, achieved a 5-month tumor-free survival (Wang et al., 2018). To increase CAR-T treatment efficacy, combining adoptive T cells therapy with chemo/radiotherapy or other target therapies are under evaluation.

In multiple aggressive solid tumors, CD133 is responsible for tumor resistance to standard therapy and tumor relapse. CD133 expression, in gastric CSCs, increases after chemotherapy treatment indicating its potential role as a therapeutic target. In a preclinical study, CD133 CAR-T cells were associated with cisplatin. This combinatorial approach inhibits *in vivo* growth, suggesting to explore the combination therapy in a future clinical trial (Han Y. et al., 2021).

### NKG2D

CAR-T cell therapy is an emerging and prominent strategy also in glioblastoma, which represents the most common brain tumors in adults with a bad prognosis. In preclinical studies, T cells engineered with a CAR that recognized NKG2D ligand, a neural stem cell marker, resulted in a safe therapeutic approach. In a study described by Weiss et al. the combination of anti-NKG2D CAR-T cells with a sub-therapeutic dose of regional radiotherapy resulted in anti-tumor synergistic activity in two syngeneic mouse glioma models (Weiss et al., 2018). However, only one clinical trial ongoing includes the treatment of refractory glioblastoma patient with the anti-NKG2D CAR-T approach alone (NCT05131763).

### ROR1

Another example is given from a preclinical study in which the combination of oxaliplatin and T cells expressing a CAR direct against tyrosine-protein kinase transmembrane receptor (ROR1) improved the anti-tumor activity of the cell therapy increasing T cell homing to the tumor site and their survival (Srivastava et al., 2021).

### GD2

All the T cell biological functions, such as cell survival, proliferation, differentiation, and cytotoxic activity, are regulated by a fine balance of specific cytokine cocktails. Modifying the cytokine network can improve CAR-T cell anti-tumor activity. Quintarelli et al., reported that the presence in culture medium of IL-7 and IL-15 increases long-term *in vitro* proliferation and survival in SHSY5Y, and IMR-32 neuroblastoma cell lines and *in vivo* expansion of CAR-T cells targeting disialoganglioside (GD2) in a NSG mice that mimic neuroblastoma (Quintarelli et al., 2018). Moreover, IL-15 significantly reduces the expression of PD-L1 on the surface of cancer cells. CAR construct was modified to express IL-7/IL-15 and the suicide gene (iC9) without impairing CAR expression and activity (Perkins et al., 2015). The iC9 gene was included in the construct as a safe strategy for the clinical application since it works as an “off switch” able to interrupt CAR-T cell cytotoxic activity at the onset of severe adverse reactions. Nowadays, CAR-T cell targeted against GD2 and expressing IL-15 and iC9 is under evaluation in a clinical trial to treat patients with neuroblastoma (NCT03721068).

### CD19

To improve the efficacy of adoptive T cell therapy, researchers are also working to increase the survival and the performance of engineered T cells. Moreover, an increased understanding of the biology of the immune system will allow the identification of targetable modulators that play a key role in T cell maturation and function (Uehara et al., 2017). Findings of a study performed by Funk et al., showed that the pre-treatment of CAR-T lymphocyte targeting CD19 antigen with a PI3K inhibitor dramatically improve CD8 T cells expansion (Funk et al., 2022). Moreover, the pre-treatment with PI3K inhibitor enhanced co-stimulatory molecules expression and the production of functional cytokines resulting in a complete tumor clearance when injected into a mouse model of human Burkitt’s lymphoma (Funk et al., 2022). *Ex vivo* expansion of engineered T cells with PI3K inhibitor could be applied to other T cell therapies.

The treatment with engineered T cell to recognize CD19 ligand has reported a complete remission rate of 54% in patients with B-cell lymphoma refractory to the standard therapy. However, many patients do not achieve complete tumor eradication after CD19 CAR-T treatment (Chavez et al., 2021). To overcome T cell therapy resistance, the CAR construct has been modified to include IL-7 and CCL-19 expression promoting T cell homing to the lymphoma tissue and enhancing cell killing activity. Moreover, patients with diffuse large B-cell lymphoma were recruited in a clinical trial in which the treatment with CD19 CAR-T expressing IL-7 and CCL-19 has been combined with PD-1 monoclonal antibody. As a consequence of PD-1/PD-L1 axis inhibition, the anti-tumor effect and long-term remission from the disease has been improved (NCT04381741).

**FIGURE 3:**
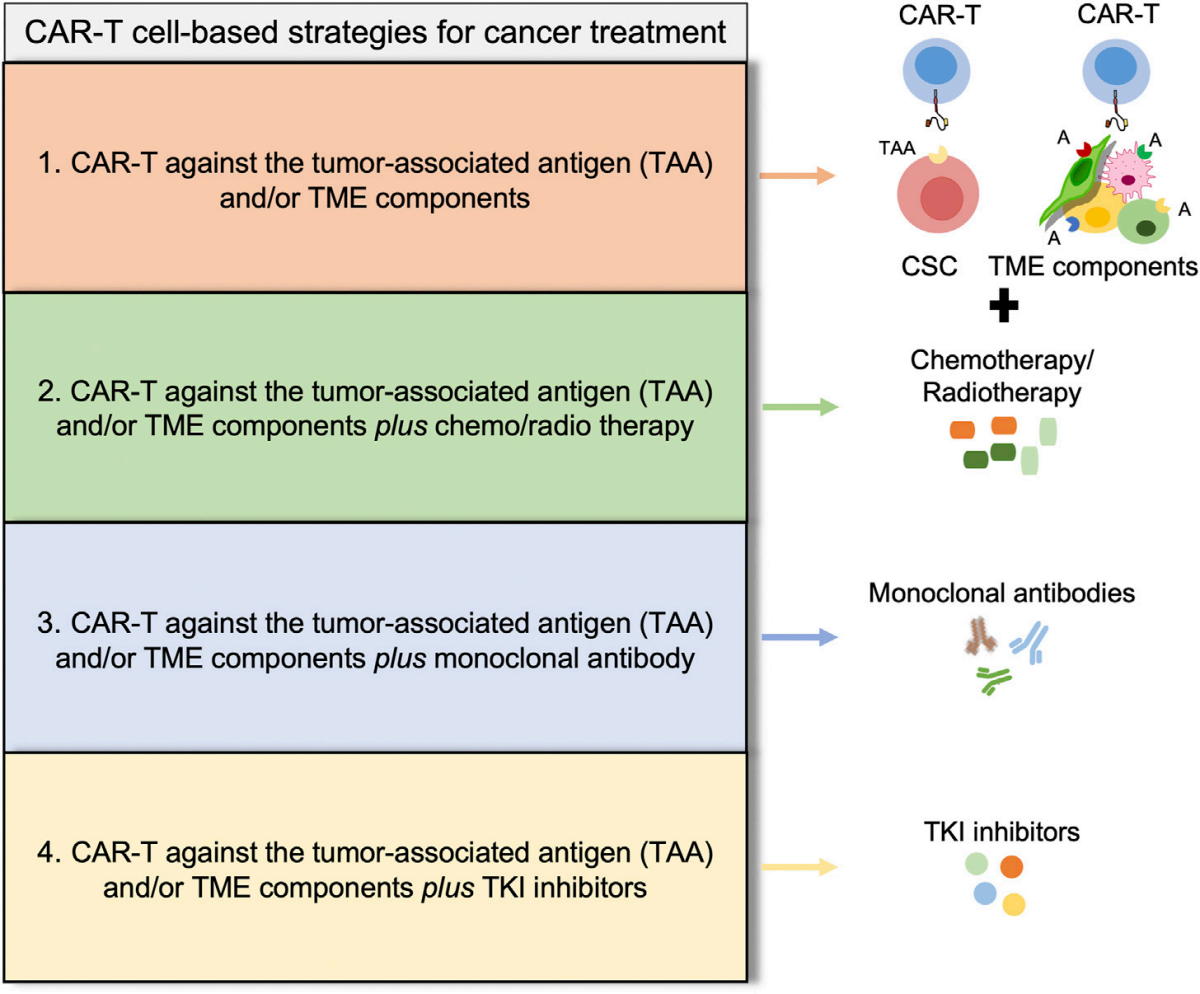
Potential strategies to overcome CAR-T cell-based therapies limitations. Illustrative scheme of different strategies for cancer treatment showing four promising approaches, discussed throughout the manuscript. (1) CAR-T cell based therapies [EpCAM CAR-T (Deng et al., 2015), CD44v6 CAR-T (Casucci et al., 2013; Porcellini et al., 2020), CD166 CAR-T (Wang et al., 2019), c-Met CAR-T (Kang et al., 2021), CD133 CAR-T (Wang et al., 2018)]; (2) CAR-T cell based therapies plus conventional drugs [ROR1 CAR-T plus oxaliplatin (Srivastava et al., 2021), CD133 CAR-T plu*s* cisplatin (Han Y. et al., 2021)]; (3) CAR-T cell based therapies plus monoclonal antibody [CD19 CAR-T plus tislelizumab (Wang et al., 2021), CD19 CAR-T plus avelumab or atezolizumab (Yamaguchi et al., 2022)]; and (4) CAR-T cell based therapies plus TKI inhibitors [PI3K inhibitor (Funk et al., 2022)]. All these alternative approaches are developed to improve the persistence of CAR-T cells and the improve immune response.

Indeed, alternative approaches have been provided to target the multiple immunosuppressive responses within TME that profoundly limit the success of immunotherapy strategies. Currently, the combination of the adoptive CAR-T cell therapy with immune checkpoint inhibitors is a promising strategy to modulate the immune microenvironment of solid tumors and increase the therapeutic efficacy of the cell-mediated anti-tumor activity. Recently, Yamaguchi et al. demonstrated that PD-L1 inhibition with atezolizumab or avelumab modulates macrophage polarization pushing toward a more M1-like phenotype improving CAR-T cell killing activity (Yamaguchi et al., 2022). An ongoing clinical trial foresees the use of CD19 CAR-T cells in combination with PD-1 monoclonal antibody (tislelizumab) for the treatment of patients with diffuse large B cell lymphoma that relapsed or are refractory to the standard therapy (Figure 3) (NCT04381741) (Wang et al., 2021).

Several clinical studies have shown that the treatment with CD19 CAR-T alone, in a large percentage of patients, cannot achieve complete remission. Considering the success of CD19 CAR-T cells as anti-tumor therapy and the expression of CD19 in the CSCs subpopulation, this specific CAR-T could be applied to target CSCs in different type of tumors.

The above discussed clinical trials have been reported in Table 1.

**TABLE 1:** Clinical trials using CAR-T cells specific for CSCs marker alone or in combinatorial therapy.

Cancer stem cells (CSCs) marker	Cancer type	NCT number	Intervention/treatment	Center	Phase	Status
**EpCAM**	Malignant Neoplasm of Nasopharynx, Breast cancer	NCT02915445	CAR-T cells recognizing EpCAM	West China Hospital	Phase 1	Unknown *
	Gastric cancer	NCT03563326	CAR-T cells targeting EpCAM	West China Hospital	Phase 1	Recruiting
	Colon, Esophageal, Pancreatic, Prostate, Gastric, and Hepatic carcinoma	NCT03013712	CAR-T cell immunotherapy	IEC of Chengdu Medical College	Phase 1 Phase 2	Unknown *
	Liver cancer	NCT02729493	EPCAM-targeted CAR-T cells	Anhui No.2 Province People’s Hospital	Not Applicable	Unknown *
	Stomach cancer	NCT02725125	EPCAM-targeted CAR-T cells	Anhui Provincial Cancer Hospital	Not Applicable	Unknown *
**CD44v6**	Cancers which are CD44v6 positive	NCT04427449	CD44v6-specific CAR gene-engineered T cells	Shenzhen Children’s Hospital, Shenzhen Geno-immune Medical Institute, Shenzhen Hospital of Southern Medical University, The Seventh Affilliated Hospital, Sun Yat-Sen University	Phase 1 Phase 2	Recruiting
	Melanoma, Breast cancer	NCT03060356	T cells modified with RNA anti-c-Met CAR	University of Pennsylvania	EarlyPhase 1	Terminated
**c-Met**	Brastcancer	NCT01837602	c-Met RNA CAR T cells	Abramson Cancer Center of the University of Pennsylvania	Phase 1	Completed
	Hepatocellular Carcinoma	NCT03672305	c-Met/PD-L1 CAR-T cell injection	The Second Hospital of Nanjing Medical University	EarlyPhase 1	Unknown *
**CD133**	Acute Myeloid and Lymphoid Leukemias, Liver, Pancreatic, Brain, Breast, Ovarian, and Colorectal cancer	NCT02541370	anti-CD133-CAR vector-transduced T cells	Biotherapeutic Department and Pediatrics Department of Chinese PLA General Hospital	Phase 1 Phase 2	Completed
**NKG2D**	Hepatocellular carcinoma, Glioblastoma, Medulloblastoma, Colon cancer	NCT05131763	NKG2D-based CAR T cells	Xunyang Changchun Shihua Hospital	Phase 1	Recruiting
**GD2**	Neuroblastoma, Osteosarcoma	NCT03721068	iC9.GD2.CAR.IL-15 T cells	Lineberger Comprehensive Cancer Center at University of North Carolina	Phase 1	Recruiting
**CD19**	Diffuse Large B-cell Lymphoma	NCT04381741	CD19–7 × 19 CAR-T plus PD1 monoclonal antibody	2nd Affiliated Hospital, School of Medicine, Zhejiang University	Phase 1	Recruiting

* Study has passed its completion date and status has not been verified in more than 2 years.

## Pitfall and critical points of CAR-T cell therapy

Although the advantages mentioned above, the use of a CAR-T cell therapy shows some limitations. A challenge not yet addressed concerns the tumor antigen heterogeneity, consisting in the expression of the different types of TAAs at different levels in tumor cell population (Chen N. et al., 2018). Strategies to overcome this limitation includes the engineering of T cells to recognize multiple TAAs simultaneously expressed on the surface of cancer cells and the use of drugs that increase the expression of CAR-T target on tumor cells (Kailayangiri et al., 2020).

Moreover, these treatment approaches counteract the escape of the tumor antigen, which is a phenomenon that has been noticed after the therapy with CAR-T based therapy. (Fry et al., 2018; Majzner and Mackall, 2018).

One of the most frequent side effects of CAR-T cell therapy is the toxicity caused by an excessive proliferation of lymphocytes, following the recognition of the cognate antigen, and the subsequent release of pro-inflammatory cytokines that characterize CRS. Several efforts have been made by the scientific community to overcome this issue. The modification of the CAR structure, in particular in the hinge and transmembrane regions have led to a reduction of cytokines levels released and a more controlled proliferation of lymphocytes, maintaining, at the same time, an excellent cytolytic capacity (Sterner and Sterner, 2021). This modification showed a favorable therapeutic response in patients enrolled in phase 1 clinical trials (Ying et al., 2019).

In addition, in solid tumors CAR-T-based therapy is limited because CAR-T cells are not able to reach and infiltrate tumor, due to the secretion of immunosuppressive factors by tumor cells and TME components (Peng et al., 2010; Moon et al., 2014). In order to overcome T cell infiltration into solid tumors, it has been tried to equip CAR-T cells with tumor-derived chemokine receptors. Anti B7-H3 CAR-T cells have been engineered to express CCL2b (Li H. et al., 2022). This construct has demonstrated to improve anti-tumor activity and enhance T cell trafficking in brain tumor lesions.

Based on this evidence, the scientific community is focusing on developing new strategies to counteract the presence of immunosuppressive factors that are released into TME, such as TGF-β. Recently, it has been developed a CAR construct directed to prostate-specific membrane antigen (PSMA) with co-expression of a dominant-negative TGF-βRII (dnTGF-βRII). The authors observed *in vitro* studies a pronounced proliferation of lymphocytes, a greater release of pro-inflammatory cytokines and a reduced depletion in CAR-T designed with PSMA with dnTGF-βRII compared to PSMA CAR-T alone. These data were further confirmed in pre-clinical models, where NSG mice treated with CAR-T PSMA with dnTGF-βRII showed stronger prostate cancer eradication than CAR-T PSMA alone (Kloss et al., 2018). A phase 1 study in patients with castrated advanced resistant prostate cancer is ongoing to define the safety and feasibility of modified autologous CAR-T PSMA-dnTGFβRII cells (NCT03089203).

Many efforts are needed to better understand the role of TME components in shaping CAR-T cell therapy response, and to develop new combinatorial strategies to overcome the above-mentioned critical points.

## Conclusion

In conclusion, the recent data collected from several pre-clinical and clinical trials encourage the adoptive cell therapy approach in the treatment of solid tumors. The association of chemotherapy and radiotherapy with CAR-T cells may improve patient clinical outcomes by acting simultaneously on the stem and differentiated cancer cells that together are the constituent of the tumor tissue. Moreover, the strategies described, to improve CAR-T cell activity by increasing lymphocyte survival and cytotoxic potential or inducing TME modulation, may be considered a striking therapeutic approach to overcome immunotherapy limitations in the treatment of solid tumors.
